# Cancer Treatment Using Different Shapes of Gold-Based Nanomaterials in Combination with Conventional Physical Techniques

**DOI:** 10.3390/pharmaceutics15020500

**Published:** 2023-02-02

**Authors:** Simona Tarantino, Anna Paola Caricato, Rosaria Rinaldi, Caterina Capomolla, Valeria De Matteis

**Affiliations:** 1 Department of Mathematics and Physics “E. De Giorgi”, University of Salento, Via Monteroni, 73100 Lecce, Italy; 2 National Institute of Nuclear Physics (INFN), Section of Lecce, Via Monteroni, 73100 Lecce, Italy; 3 “Vito Fazzi” Hospital of Lecce, Oncological Center, Piazza Filippo Muratore 1, 73100 Lecce, Italy

**Keywords:** cancer, diagnostics, medical physics, therapy, nanomedicine, gold nanoparticles, nanostars

## Abstract

The conventional methods of cancer treatment and diagnosis, such as radiotherapy, chemotherapy, and computed tomography, have developed a great deal. However, the effectiveness of such methods is limited to the possible failure or collateral effects on the patients. In recent years, nanoscale materials have been studied in the field of medical physics to develop increasingly efficient methods to treat diseases. Gold nanoparticles (AuNPs), thanks to their unique physicochemical and optical properties, were introduced to medicine to promote highly effective treatments. Several studies have confirmed the advantages of AuNPs such as their biocompatibility and the possibility to tune their shapes and sizes or modify their surfaces using different chemical compounds. In this review, the main properties of AuNPs are analyzed, with particular focus on star-shaped AuNPs. In addition, the main methods of tumor treatment and diagnosis involving AuNPs are reviewed.

## 1. Introduction

Cancer is the second leading cause of death in the world. In recent years, nanotechnology has become an integral part of medicine field, allowing researchers to make significant developments regarding tumor diseases. Among the different kinds of nanomaterials, AuNPs are the mostly used ones due to their biocompatibility and their unique physicochemical properties [1]. These properties, including high surface-area-to-volume ratio, small size, chemical stability, and Localized Surface Plasmon Resonance (LSPR), differ from those of the bulk material [2].

LSPR is a typical phenomenon of metallic NPs, and it consists of the interaction between the NPs and the light of resonant frequency, causing plasmonic oscillations. These oscillations can be kept within a wide spectral range from the visible to the near-infrared regions (NIR). This occurs when the NPs’ shape deviates from a symmetrical nano spherical one to those of nanoshells, nanocages and nanorods [3]. Therefore, since the LSPR phenomenon is dependent on the particle size, shape, and structure of the specific metal NP [4], specific and reproducible synthetic approaches, which are performed to control the AuNPs’ size and shape [5], are fundamental to tune the optical properties of AuNPs [6,7]. For example, spherical AuNPs, silver nanoparticles (AgNPs) and copper NPs (CuNPs) exhibit strong LSPR bands in the visible region. The hollow or core–shell NPs induce a red-shifted band of the LSPR wavelength, while other metals show weak bands in the UV region [4]. All of this makes the use of metallic NPs, rather than non-metallic NPs, extremely important. In fact, the non-metallic NPs, do not exhibit the LSPR phenomenon, thus making them not useful for multiple cancer treatments exploiting different types of radiation.

Thanks to their high surface area, AuNPs can be functionalized with different moieties depending on their use. Then, the surface decoration by antibodies, small molecules, drugs, hormones, or peptides drives the NPs to specific target the areas exposed on the cell membrane. For example, several peptides for AuNPs functionalization, such as arginyl-glycyl-aspartic acid (RGD) (Arg-Gly-Asp), have been used to target pancreatic cancer cells. In this case, thanks to integrin receptors on the surface of many tumor cells, receptor-mediated endocytosis occurs, and the RGD-functionalized AuNPs easily enter the cytoplasm area [8]. About targeting using antibodies, Cruz et al. [9] experimented with an antibody–drug conjugate (ADC) used as a targeting agent–drug carrier on the AuNPs. They synthesized 50 and 20 nm Trastuzumab-conjugated AuNPs (Tmab-PEG-AuNPs) for use with SKBR-3 cancer cells (HER-2 positive). The levels of the targeting and internalization activities at the tumor site were significantly higher than those of the simple OH-PEG-functionalized AuNPs.

Instead, AuNPs can be functionalized with biocompatible polymers to improve their ability to circulate in the blood stream, enhancing their half-life and avoiding their expulsion [10]. Among these, there are the poly(lactide) (PLA), poly(lactide-co-glycolide) (PLGA) copolymers, poly (ɛ-caprolactone) (PCL) and poly(amino acids). If, on the other hand, natural polymers are used, then a reference is made to elements such as chitosan, gelatin and albumin [11]. This is a critical aspect for drug delivery applications. 

Furthermore, from recent studies, AuNPs are proving to be excellent tools as chemotherapeutic agents in the cases of therapy resistance [12]. Both unmodified and modified AuNPs have been shown to inhibit cell proliferation and increase sensitivity to cisplatin (Pt^II^) and gemcitabine (GEM) in several cancer models. 

Huai et al. [13] examined if the AuNPs triggered the clonal growth of pancreatic ductal adenocarcinoma (PDAC) cells and, at the same time, sensitize them to GEM. They performed two-dimensional (2D) and three-dimensional (3D) sphere formation assays and treated them with different doses of AuNPs (20 nn) (5 µg/mL, 10 µg/mL and 25 µg/mL). In the 2D case, three AuNPs doses decreased the colony numbers of PANC-1 cells by 43%, 82% and 99%, respectively, and in MIA PaCa-2 cells, they decreased it by 5%, 28% and 60%, respectively. Instead, the pancreatic cells line AsPC-1 did not undergo any alteration. In the 3D case, the three concentrations of AuNPs decreased the colony numbers in PANC-1 cells by 25%, 50% and 70% and by 20%, 40% and 50% in AsPC1 cells and by 15%, 30% and 70% in MIA Paca 2 cells, respectively. Regarding the sensitization of pancreatic cells by AuNPs, they considered four pancreatic cell lines: AsPC-1, PANC-1, MIA PaCa-2 and HPAF II. The cells were exposed to AuNPs for 24 h, and then with GEM for 72 h. It turned out that cells pretreated with AuNPs required much less GEM than the treatment with GEM alone did. In particular, they exhibited a half-maximal inhibitory concentration of GEM, the amount of which was 17 times, 7.4 times, 2.1 times and 6.7 times smaller in the four cell lines, respectively. 

Cisplatin-complexed AuNPs have been investigated by Tan et al. [14] to improve targeting and enable high delivered doses of Pt^II^ to cancer cells, thereby preserving healthy tissues. They synthesized Pt^II^ complexed to SH-PEG and thiolated carboxyl-terminated dendron (AuNP-(dendron)-(SH-PEG)). The NPs (size 15 nm) were incubated in an environment with a pH that is equal to five in order to mimic the acidic pH of the lysosomal compartment of a tumor. AuNP-(dendron)-(SH-PEG)-high Pt^II^ (C_Pt_^II^ = 11.4 µmol/L) and AuNP-(dendron)-(SH-PEG)-low Pt^II^ (C_Pt_^II^ = 1.1 µmol/L) were compared. The amount of Pt^II^ that was released was six time greater in the high Pt^II^ condition than it was in the low Pt^II^ condition.

All of these characteristics make AuNPs suitable for several biomedical applications such as diagnosis and therapy. The diagnostic AuNPs-based techniques include computed tomography (CT), photoacoustic imaging (PAI), single-photon emission computed tomography (SPECT), positron emission tomography (PET) and magnetic resonance (MR). The therapeutic applications using AuNPs include radiotherapy (RT), photothermal therapy (PTT), photodynamic therapy (PDT), chemotherapy and gene therapy [15].

In this review, we first describe the characteristics and properties of the nanoscale material, as opposed to those of the material in bulk form, focusing on AuNPs with different shapes, including nanospheres, nanorods, nanowires, nanoprisms and nanoshells. In the second part, the description of star-shaped AuNPs properties are fully discussed. In the last two sections, the attention is focused more on the advantages and benefits deriving from the use of AuNPs in the medical field in two big areas, diagnostics and tumor therapy, illustrating the working principle of their traditional use and commenting on the improvements after the AuNPs applications. About tumor therapy, three methods are reported and commented on: RT, PTT and PDT. Instead, for diagnostics we include the following methods: CT, PAI and SPECT. 

## 2. Physicochemical Properties of Au at Nanoscale

Nanomaterials are defined as objects with a size of less than 100 nm. The ability to “separate, consolidate and deform materials starting from an atom or a molecule” [16] is called nanotechnology, which was first coined in 1974 by the Japanese engineer, N. Taniguchi.

In recent decades, the world of nanotechnology has aroused considerable interest in the scientific community. The attention of scientists is attracted by the properties (physical, chemical, electrical, mechanical and optical ones) possessed by the nanostructures compared to those of the same material in the bulk form. For example, some factors and/or interatomic interactions that have no relevance at the macroscopic level play a fundamental role when we are considering the nanoscale dimension [17].

Generally, two approaches are used to obtain nanomaterials: the ‘top-down’ approach, where bulk materials are broken down to the nano size by milling or etching, and the ‘bottom-up’ approach, which makes nano-sized objects by combining atomic scale materials [18].

**Figure 1:**
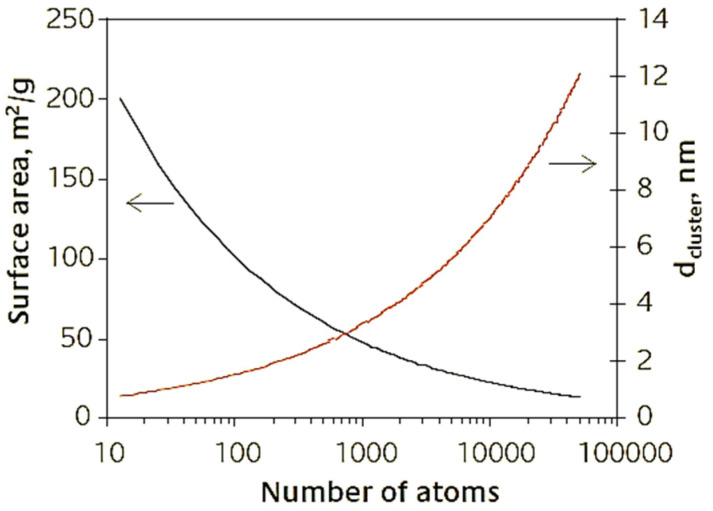
AuNPs atoms number as a function of surface area and size [17].

**Figure 2:**
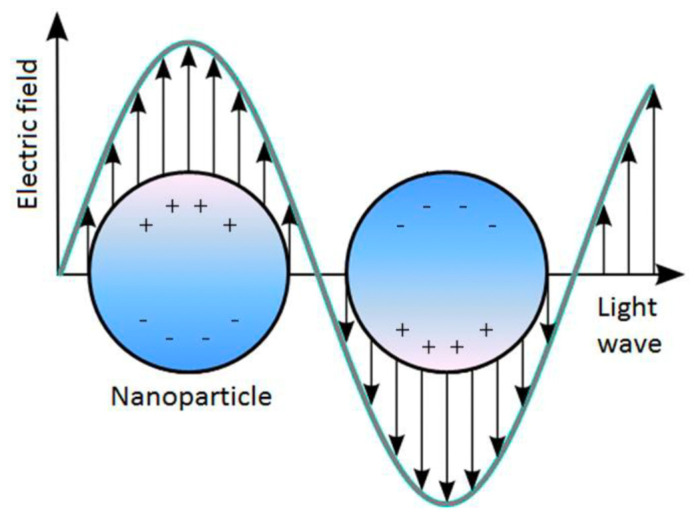
LSPR effect: plasmon oscillation for a AuNP [20].

**Figure 3:**
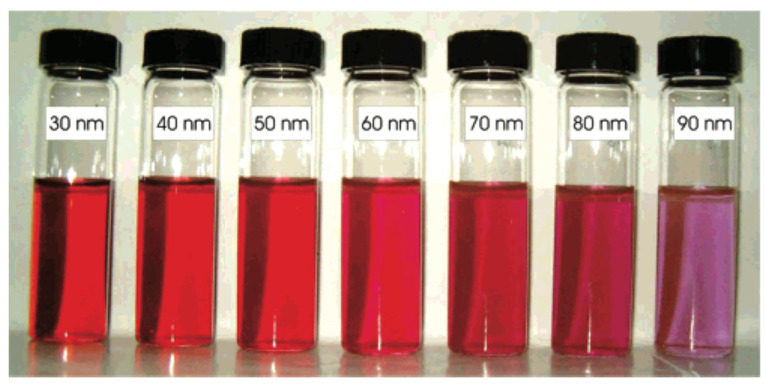
Different sizes of spherical AuNPs correspond to different color solutions. Reprinted (adapted) with permission from [24]. Copyright 2007, American Chemical Society.

**Figure 4:**
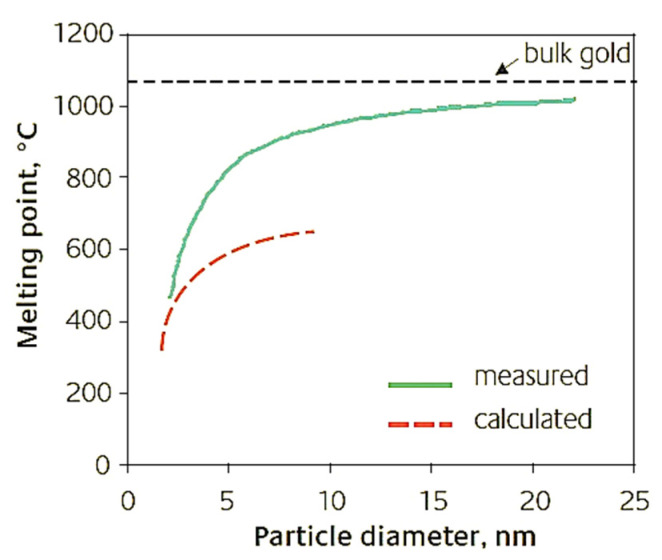
Cartesian graph of the different values of the melting point vs. AuNPs’ dimensions [17].

Nowadays, among the different types of materials, Au has innumerable properties when it is reduced to nanometric dimensions, in particular:

- Their small size increases the number of surface atoms compared to the number of those in the volume. This happens because as the size decreases, there is a large increase in the surface/volume ratio (Figure 1). For this reason, any chemical reaction or phenomenon that occurs on the surface is enormously amplified [17]. Furthermore, the surface/volume ratio increase is responsible for the chemical and physical differences that nanomaterials possess that the bulk material does not.
  The high surface/volume ratio has some rather important implications for the behavior of the nanostructures themselves. If a bulk material is divided into smaller pieces, the volume remains constant, but the size of the surface undergoes a significant increase. Since each surface is associated with a specific surface energy, E_sup_, there will be an increase in the total E_sup_ of the system. For this reason, nanostructured materials are metastable or thermodynamically unstable. This scenario explains why NPs have a strong tendency to agglomerate.
  To avoid agglomeration, some precautions can be adopted during the synthetic stage. These include the use of surfactants that work by adsorbing onto the AuNPs’ surface, increasing the repulsion forces among the NPs, thus preventing agglomeration.
  Controlling parameters such as temperature and synthetic times may possibly reduce the degree to which the AuNPs clump.
  Finally, an additional expedient is to use several AuNPs washes once they are synthesized. This allows them to be purified from organic and/or inorganic residues due to the presence of solvent materials used during the synthesis phase [19].

- In addition, Au is a noble metal material that has unique optical and electrical properties that are dependent on the LSPR phenomenon, which is typical of metal NPs. It refers to an oscillation of free electrons on the interface of the metal surface produced after the interaction with an incident electromagnetic wave. In particular, it happens if the frequency of the electromagnetic wave resonates with that of the electrons in the conduction band, generating a plasmon band (Figure 2).
  The result is an intense diffusion and strong absorption of light, combined by an enhancement of the electric field near the nanostructure surface [21,22].
  The LSPR phenomenon is more effective in Au, as well as more sensitive. Furthermore, its properties make it suitable for the biosensor’s development or as therapeutic tool, which exploit the phenomenon described above.

- The color of Au at the nanoscale is different from that of the material in the bulk form.
  At the macro scale, Au is yellow, with differences based on the brightness, depending on the surface roughness and on the purity of the material. When the size is reduced to the nanoscale, the color considerably changes due to the LSPR effect.
  For small, spherical Au nanostructures, a strong plasmon absorption band is observed at 520 nm [23], resulting in a burgundy color. By varying the shapes and sizes, the absorption spectra are shifted. Generally, the color varies from light violet to burgundy to blue (Figure 3).

- Under standard conditions, the melting point of Au in the bulk form is ∼1064 °C. As the size of the AuNPs decreases to below 10 nm, the T_fus_ also decreases. This is due to the high surface/volume ratio.
  Below, the different melting points calculated by Dick et al. [25] and Liu et al. [26] (Table 1) and the related cartesian graph (Figure 4) are reported.

**Figure 5:**
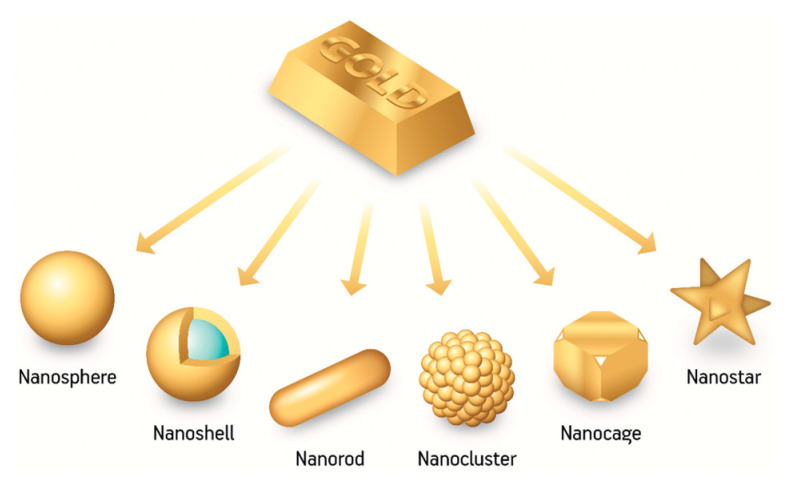
Different AuNP shapes [28].

AuNPs can be obtained from different synthesis procedures that allow us to modulate their size and shape [27]. The most common shapes are nanospheres, nanorods, nanoprisms, nanowires, nanocages and nanostars (Figure 5).

Au is also low toxic, even if its long-term effects on humans are not fully understood [29].

## 3. Why Au Star-Shaped NPs?

**Figure 6:**
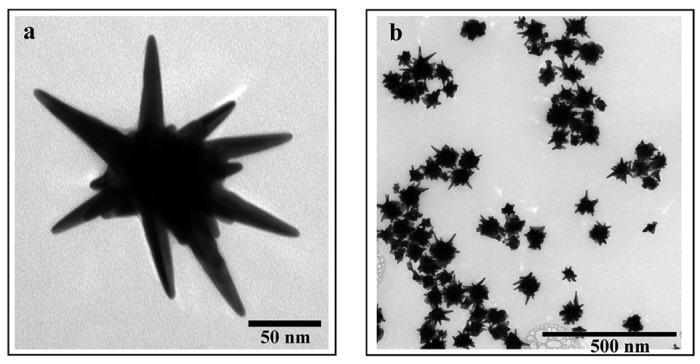
AuNSs images acquired by Transmission Electron Microscopy (TEM) at different magnifications: 50 nm scale bar (**a**) and 500 nm scale bar (**b**) [30].

Since AuNPs exhibit specific physicochemical properties based on their shape, a lot of different forms are produced such as nanorods, nanoprisms, nanodimers, multi-spikes and AuNSs. AuNSs’ properties depend on their structure, which can be made of cores and sharp apexes on the surface (Figure 6).

The LSPR phenomenon in AuNSs is controlled by the cores’ and spikes’ dimensions [31]. Thanks to the large extinction cross section of the AuNSs, their interaction with electromagnetic waves is extremely intense, thus ensuring the interaction of light with each individual particle [32].

In contrast to spherical AuNPs that absorb only in the visible region, anisotropic AuNPs (i.e., nanoshells, nanorods, nanocages, etc.) have an absorption band in the NIR range [33]. However, among the several anisotropic shapes, the AuNSs exhibited the greatest absorption in the NIR range, which makes them suitable for use in cancer treatments as a photo-thermal therapy (PTT). 

Pakravan et al. [34] investigated the effect of seven AuNP shapes on their optical properties and their efficiency in a photo-thermal process, in which AuNPs generate heat by laser irradiation. They synthesized hollow, rod, cage and sphere AuNSs with Fe-Au and Si-Au cores. Both the absorption and the photo-thermal conversion efficacies of the seven AuNPs shapes (all at the same concentrations) were compared by calculating the temperature increase for their aqueous suspensions at a 0.7 W/cm^2^ laser power density under 808 nm NIR light for 10 min. After irradiation, the lowest photo-thermal conversion efficiency was recorded for the nanospheres (38 °C), followed by the Fe-Au NPs (47 °C), Si-Au NPs (48 °C), Au nanocages (49 °C), Au nanorods (50 °C) and Au nano hollows (52 °C). The best conversion was performed by the AuNSs, with a final temperature of 53 °C. This study demonstrated that geometry affects the NIR absorption and conversion efficiency, making the AuNSs promising candidates for NIR cancer treatment. 

This is particularly interesting because the biological tissues are quasi-transparent in the near-infrared (NIR) region (700–900 nm) [35], and so they are fundamental for applications in the biological field [36]. 

However, by distinguishing AuNSs from other non-spherical AuNPs, a significant discrepancy is observed in terms of the resonance shift. In fact, for the AuNSs, the extinction spectra show peaks between 1000 and 1400 nm thanks to the presence of spikes. On the other hand, for all of the other AuNP shapes, the absorption peaks do not exceed the 1000 nm wavelength. As opposed to other Au nanostructures, the AuNSs’ structure consists of a circular structure with a sharp tip, providing specific regions called “hot spots” at the sharp ends. This feature provides optical enhancement when specific wavelengths interact with them, such as NIR or X-rays [37]. The AuNSs are ideally suited to applications of surface-enhanced Raman spectroscopy (SERS). SERS allows researchers to analyze the structural properties of single molecules thanks to the interaction between a laser light and the matter, exploiting the usefulness of plasmonic NPs [38]. 

The difference between Raman spectroscopy and SERS is that SERS requires the presence of a metallic nanostructure [39], thus noble metal NPs are used as substrates [40]. 

In Raman spectroscopy, a laser source applies incident light to a chemical sample. Part of the incident light is absorbed, while a small part is inelastically scattered at different wavelengths, acting as a fingerprint for the vibrational transitions of the molecule. Despite its specificity, Raman spectroscopy signals are too weak, thus SERS is beginning to be considered as the correct technique for the analysis of molecules [41].

The metal NPs used as a substrate in SERS permit both chemical and electromagnetic enhancements, then stronger signals. The chemical enhancement is due to the occurrence of a Raman transition when the molecules are adsorbed on a roughed surface, while the electromagnetic one is due to the LSPR phenomenon [42]. Then, the local electric-magnetic field generated is ten orders of magnitude greater than the incident one, and therefore, it is extremely useful for SERS applications [43].

The total electric field generated strongly depends on the size of the NP, its shape and its orientation with respect to the polarization of the excitation light. AuNSs are considered to be the best nanostructures for SERS applications.

## 4. In Vitro and In Vivo Applications of AuNPs for Cancer Therapies

Cancer is the second leading cause of death in the world. Genetic mutations, i.e., alterations to cell DNA, together with their uncontrolled proliferation, trigger malignant progression and metastatization phenomena. The genetic alterations can include random, systemic and metabolic ones that are very difficult to manage. This multi-factorial profile makes cancer difficult to treat [44]. The key element for the success of any therapy is early diagnosis, rather than prevention [37].

The medical and technological developments in recent decades have allowed us to obtain a great amount of control of tumor diseases, both at the diagnostic and at the therapeutic levels.

Today, the main tools used to reduce and/or inhibit tumor progression are radiotherapy, chemotherapy and surgery [45].

In conventional radiotherapy, ionizing radiations, X-rays or γ-rays of the order of MeV, are used in order to damage the DNA of the malignant cells, causing apoptosis and necrosis, and finally, death. 

In chemotherapy, chemotherapeutic agents such as drugs are administered to stop the activity of tumoral cells based on their toxicity [46].

Surgery, as well as radiotherapy, is fundamental when the solid tumor is confined to a specific organ and metastases have not developed in other tissues [47].

However, each mentioned treatment has limitations that can hinder its efficiency over time. Chemotherapy, for example, has severe consequences that are classified by the World Health Organization (WHO) as mild (grade 1), moderate (grade 2), severe (grade 3) and disabling (grade 4) ones [48]. These include the most nausea, vomiting (even more than ten times a day) and damage to the bone marrow, blood, kidneys and intestines [49]. Other limiting factors for the aforementioned tumor therapies are multi-drug resistance (MDR) due to the repetitiveness of chemotherapy or radiotherapy treatments over time [50], the heterogeneity of the dose delivered to the patient, the unavoidable dose of radiation also for healthy tissues [51], cardiac complications [52] and pneumonitis [53].

The study of metal nanomaterials represents a promising opportunity for diagnosis and therapy. In particular, AuNPs have begun to be used in radiotherapy as radiosensitizers, thereby attracting the attention of the scientific community [54].

For example, the easy surface functionalization of AuNPs by thiols chemical groups is a valid strategy to introduce reactive functional groups that are useful for targeting and drug delivery [55]. In this way, thanks to the AuNPs–drugs bond, the efficacy of the drug is increased, targeting the cancer cells directly and allowing their correct absorption by the tumoral cells. Additionally, modifying the AuNPs’ surface with certain antibodies further improves the specificity of the drug delivery [56].

In the following paragraphs, the three major AuNPs-based physical approaches used to treat tumor cells are described.

### 4.1. Radiotherapy

**Figure 7:**
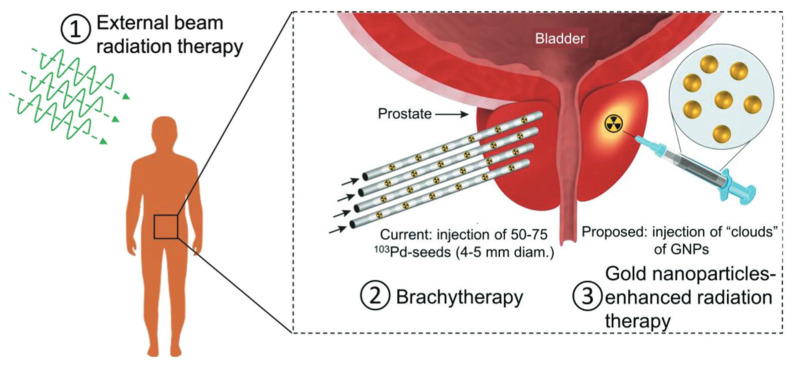
Three possible radiotherapy approaches: (1) External beam radiation therapy; (2) brachytherapy; (3) AuNPs-enhanced radiation therapy. Reprinted (adapted) with permission from [58], John Wiley and Sons.

During a conventional radiotherapy treatment, the patient is subjected to megavoltage (MV) X-rays beams. The principle based on this treatment is that cancer cells are more sensitive to the DNA damage response than normal cells are [57] (Figure 7).

Radiation therapy uses high-energy radiation in the form of waves (such as X-rays) or particles (such as protons) to kill cancer cells or stop them from growing and spreading. The X-ray energies are used in the range of between 80 keV and 25 MeV, generally in the 8–18 MeV range, using modern high-energy linear accelerators that produce the radiation beam [59].

However, there are several concerns regarding the outcome of the treatment, which depend in turn on the tumor radiation resistance and radiation absorption coefficient. In general, the radiation can be applies using different techniques such as an external beam (electrons, photons or protons) or an internal radioactive source (brachy therapy), but the beam can be directly induce DNA damage (direct action). Alternatively, a genetic break can occur by the activation of reactive oxygen species (ROS), namely ·OH, NO·, H· and H_2_O_2_, due to secondary electrons and the direct fragmentation of the DNA and apoptosis (indirect action). However, when external beam radiation is used, healthy tissues are also exposed to radiation, increasing the normal tissue complication probability. Indeed, several epithelial–mesenchymal transition (EMT) transcription factors can be activated, promoting cancer cell metastasis [60].

More than 50% of individuals with cancer undergo radiotherapy, and they are aware of the risk of late cardiovascular morbidity [61] and the development of secondary tumors. These are more likely to develop at least in the decade following treatment, depending on the dose of the radiation, which affects healthy tissues and the anatomical area treated during radiotherapy [62]. Younger patients have a higher risk of developing secondary cancers [63]. For example, the risk of thyroid cancer occurring is more likely in individuals undergoing radiotherapy for the neck region [64,65]; rectal cancer is a consequence for those who undergo X-radiation for prostate cancer [66].

In this context, nanotechnology can be a valid tool to overcome a lot of disadvantages connected to radiotherapy, improving the therapeutic efficacy. In addition, the resistance of tumor cells to radiation means that they may require a high therapeutic dose, which is dangerous to normal tissues and cells, whereas nanomaterials offer the possibility to reduce the side effects on the healthy tissues [67].

The need to balance the benefits and the side effects of the radiotherapy means that researchers must integrate the use of metal nanomaterials and the radiotherapy treatments. They act as “radiosensitizers”, concentrating the radiation dose delivered to the tumor cells, thus sensitizing them. In this way, the healthy tissues surrounding the tumor are preserved as much as possible. 

Then, the use of radio sensitizers is important to enhance the efficiency of radiotherapy, but adverse effects have been recorded. In this context, the application of AuNPs can be particularly suitable to increase and improve the efficiency of radiotherapy due to their high atomic number (Z = 79) and density (=19.3 g/cm^3^), which are crucial for tissue damage. As matter of the fact, the high number of Z materials can strongly absorb more energy per unit mass (10–150 times for kV photons) than other soft tissues can [68].

AuNPs have a cross section of interaction with X-rays and with ionic radiation of up to about 1 MeV [46].

The efficacy of radiotherapy in the presence of AuNPs can be measured by the dose enhancement factor (DEF). This factor related to AuNPs is expressed as the ratio of the radiation dose absorbed by the tumor cells to that which is absorbed without the presence of AuNPs [69,70]. The value depends, in turn, on certain factors such as the concentration of the NPs and their chemical and physical properties, as well as their intracellular localization.

Some Monte Carlo simulations performed by Zhang et al. [71] showed that the radiation beam on cells after AuNPs internalization resulted in the increase in the therapeutic dose using a lower dose of radiation. 

Of course, the effectiveness of the treatment using AuNPs depends on their concentrations, size and shape. The interaction between NPs and X-rays/γ-rays is explained through the phenomenon of electron excitation and scattering on AuNPs [72].

When the excited electron comes from an inner shell, Auger de-excitation processes involving Auger cascades, i.e., the emission of many electrons, even more than 10, are likely to occur [73]. These electrons are capable of damaging DNA while ionizing the water molecules they encounter in their path, with an energy of less than 5KeV.

Because NPs are found in a conspicuous number within a cell after uptake, all of the secondary electrons can in turn interact with other NPs, causing other Auger electron emissions. In addition, they can also result in the formation of radicals when they are absorbed by the medium instead.

The high radio sensitization of AuNPs that is observed when they are hit by photons can be explained by taking into account a high cross-sectional area up to the megavolt range with respect to biological matter such as cells, which are composed mostly of water. This characteristic allows researchers to achieve greater localization of the dose that is administered.

As per the photoelectric effect, which scales proportionally to (Z/E)^3^, where Z is the atomic number of the element and E is the energy of the photon, the previous described emission, i.e., Auger’s emission, occurs to a greater extent when the atomic number Z of the elements is high, as is true in the case of Au [74]. This emission is greater compared to those of the elements with a lower atomic number. More specifically, this is the case with C, O and H, which make up most biological molecules. The increment of the Au cross section relative to that of other atoms tends to decrease if high energies are used. Some studies have highlighted the great peculiarity of Au in combination with radiation, in fact, it was reported that when one was using AuNPs and low-energy X-rays (80 kVp), a high DEF was found; the values were proportional to the concentration of NPs that were used. By using a 1 mM concentration of AuNPs, the DEF was 24.6, which was compared to a value of 4 that was measured using a concentration of 0.25 mM [75].

In addition to the good physical properties of AuNPs, they are biocompatible and inert, thus they do not react with other elements within the biological environment, showing lower toxicity compared with that of elements such as cisplatin (chemotherapeutic agent) or nitroimidazole. In addition, they exhibit low systemic clearance, which allows the AuNPs to be confined to the tumor tissue, sensitizing it adequately. This is also due to the enhanced permeability and retention (EPR) effect, favoring the deposition of AuNPs at the tumor site. The high surface/volume ratio allows the therapeutic agents such as proteins, antibodies, or drugs to bind to the surface of the nanoparticle for a personalized treatment [51,55,76,77].

The first fundamental in vivo experiment, in which AuNPs were used as active tools in radiotherapy, is showed in the work of Hanfield et al. [78]. AuNPs (size: 1.9 nm) were administered by injection to mice with subcutaneous EMT-6 mammary carcinomas. Each mouse received a single intravenous dose of 1.35 g Au/kg AuNPs. The tumor region was irradiated with 250 kVp X-rays. Comparing the results, the survival rate among mice given the radiotherapy without AuNPs was 20%. Instead, a combined AuNPs and radiotherapy treatment showed a 50% survival rate in the animal models. This result was further compared to that of a group of mice given AuNPs, but without radiotherapy. In the latter case, no animal survived [78].

In 2013, Bobyk et al. [79] analyzed the effect of AuNPs in rats with brain tumors. They were treated with 5 microliters of 15 nm AuNPs (50 mg/mL) by intracerebral injection 11 days after the implantation of 4000 F98 cells. After twenty minutes, the rats were irradiated with 88 keV X-rays. The rats that did not receive any treatment lived, on average, for 23.8 ± 1.6 days; the rats that received only the AuNPs without irradiation lived for 23.3 ± 0.7 days; the rats subjected only to X-radiation survived for 33 ± 2.7 days; finally, the rats subjected to an injection of AuNPs and irradiation lived for 41.6 ± 3.2 days [77,79].

Teraoka et al. [80] studied the effect of AuNPs in radiotherapy with an in vitro experiment using a human head and neck carcinoma cell line, HSC-3. The tumor cells were treated with 5 nm AuNPs at four different concentrations, i.e., 0.1 nM, 0.4 nM, 1.0 nM and 10 nM. X-ray irradiation was performed at three doses of 2 Gy, 4 Gy and 8 Gy. The number of cells decreased until there were 50% of them, especially using 4 Gy and 8 Gy irradiation doses.

In addition to the radiosensitization induced by spherical AuNPs, nanoparticles of different shapes, such as nanostars, were also analyzed. As already discussed above, the AuNPs subjected to irradiation involve the emission of Auger electrons responsible for damaging cancer cells [81]. Furthermore, Sicard-Roselli et al. showed that AuNPs, especially star-shaped ones, act as radiosensitizers thanks to the increase in the hydroxyl radical, which is able to react strongly with critical cellular biomolecules such as DNA, inducing cell death [81,82]. In particular, when they were using the 2 M AuNPs concentration, the amount of OH radicals was greater in the presence of star-shaped NPs than it was with the triangular or spherical ones. This was measured after the incubation of the γ-irradiated aqueous solution of these NPs with oxidation-sensitive fluorophore. In order to correlate the radio-sensitizing effect with the production of OH radicals, adenocarcinomic human alveolar basal epithelial cells (A549) were treated with star-shaped, triangular and spherical AuNPs (size: 60 nm, and concentration: 5 nM). After γ-irradiation, the treatment with AuNSs was more efficient than the others were. It is linked to the capacity of AuNSs to produce OH radicals after γ-irradiation in a better manner compared to the other Au shapes [81].

### 4.2. Photo-Thermal Therapy (PTT)

Conventional cancer therapies, such as radiotherapy or chemotherapy, not only damage the cancer cells, but they also damage the surrounding healthy biological tissues. A further disadvantage of these treatment methods is the possibility of developing drug resistance or radio resistance, making therapeutic success highly unlikely.

The use of heat in tumor therapy (hyperthermia) is a promising tool due to the sensibility of malignant cells to high temperature in the range of 41 ÷ 47 °C for a few minutes of exposure [83]. This heating irreversibly damages the cancer cells because of their low heat tolerability due to their higher metabolic rates [84]. However, a non-negligible aspect of hyperthermia is the damage to healthy tissues and the denaturation of proteins that are near the cancer area.

An innovative tool for cancer treatment is the use of specific laser sources in order to reduce the damaging exposure of non-cancerous tissues. 

Photo-thermal therapy (PTT) requires 700–1100 nm of the near-infrared (NIR) wavelength using a laser in combination with therapeutic agents capable of absorbing the incoming wavelength and propagating it to tissues and local tumor cells in the form of heat [85].

However, the complete eradication of each cell occurs when the temperature almost 50 °C [86]. In particular, the cell death pathway is distinguished in apoptosis and necrosis [87,88,89]. The first one occurs at around 44 °C, causing an orderly and regular type of death, with the consumption of ATP; the second one, which occurs at temperatures higher than 46 °C, causes cellular trauma or large amount of stress [87,90].

Although the NIR laser beam is characterized by high spatial precision and strong intensity, its low absorption within the tumor site makes the use of therapeutic agents in PTT inevitable [91]. In this context, the AuNPs represent an excellent ally for photothermal therapy. Thanks to their enhanced absorption cross section, AuNPs absorb up to five orders of magnitude more light than the other photoabsorbent dyes do. Then, on a time scale of a picosecond, they convert the light into heat in order to kill the cancerous cells irreparably [92]. In particular, thanks to surface plasmon resonance, every AuNP shape absorbs light in the NIR range in a different way [84]. As described above, anisotropic AuNPs exhibit the highest absorption rate in the NIR, greatly enhancing the cancer cells’ destruction. By altering the shape and size of the AuNPs, it is possible to tune the resonant frequency for absorbing the light of a particular wavelength [86]. Regarding the size, smaller AuNPs are associated with better efficiency in the conversion of absorbed light into heat. They have a better absorption ability and higher photothermal efficacy because they require much less laser energy than larger AuNPs of the same type do to destroy a specific tumor [93]. On the other hand, anisotropic AuNPs, besides having a higher absorption rate in the NIR, are capable of converting the light into heat with high efficiency. In particular, AuNSs exhibit a record light-to-heat conversion efficiency due to the presence of spikes [94].

The first use of Au nanoshells, i.e., nanomaterials consisting in a dielectric core (often silica) and a thin metallic shell, usually Au, in photothermal therapy was described in 2003 by Hirsch et al. [95]. The plasmonic absorption can be tuned to the NIR region by modifying size and shape of the NPs [87].

Increasing the size of Au nanospheres, Au nanorods and silica-Au nanoshells induces an increasing extinction cross section [96]. Consequently, the surface plasmon absorption redshifts. 

**Figure 8:**
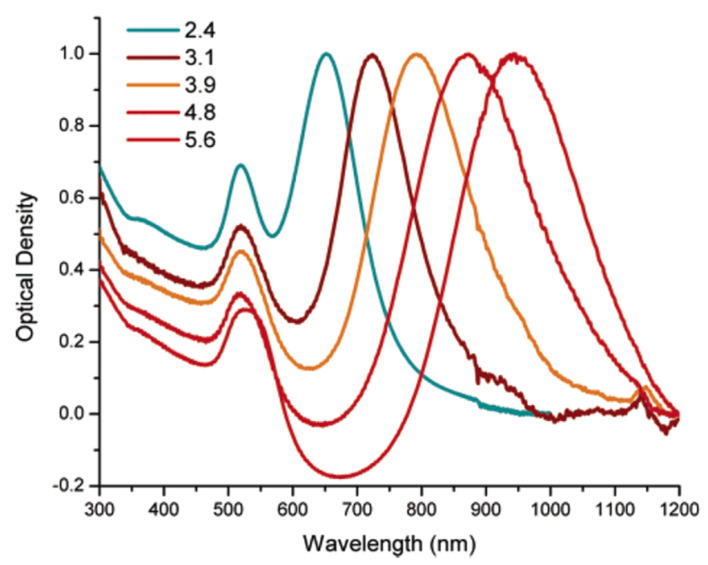
Surface plasmon absorption spectra for different nanorods’ aspect ratios. Reprinted (adapted) with permission from [97], Copyright 2006 American Chemical Society.

Huang et al. [97] exploited the property of noble metals to generate strong electric fields on the surface of the NPs. In this way, the absorption and scattering of the incident electromagnetic radiation is also significantly increased. This aspect is very useful for PTT. They have synthesized Au nanorods with different aspect ratios to absorb radiation in the NIR range. After irradiation with a laser at 800 nm, both malignant, i.e., two oral squamous carcinoma cell lines (HSC *313* and *HOC* 3 *Clone 8*)*,* together with non-malignant cells, i.e., keratinocytes (HaCat), were photothermally destructed. In addition, the longitudinal absorption band also increases to the NIR region, thereby increasing the aspect ratio of the nanorods (Figure 8).

The study revealed that, in this case, to destroy malignant cells, nanorods need less energy (about half) than it takes to destroy healthy cells.

AuNP_S_ are characterized by thermal stability, therefore, their properties remain unaltered even at high temperatures [98], and by the ability to spontaneously reach the tumor site and accumulate in the malignant vascularized area (enhanced permeability and retention (EPR) effect) [55].

Moreover, there are other factors that define the efficiency of PTT. First, the AuNPs are more able to convert light into heat when they are small [99]; by increasing the irradiation time, the number of killed cells increases proportionally [100]. In addition, the increase in the NPs concentration cause a greater efficiency of the PTT [101,102].

The phenomenon of AuNPs hyperthermia can be used also in combination with other therapeutic approaches (radiotherapy, chemotherapy, immunotherapy, etc.) to enhance its efficacy. Hainfeld et al. [103] evaluated the synergy of AuNPs hyperthermia and radiotherapy on a very radioresistant subcutaneous cell carcinoma in mice. They synthesized -AuNPs with a diameter of 15 nm, which were aggregated in endosomes and lysosomes. This expedient was adopted because bare, spherical AuNPs absorb only ultraviolet (UV) and visible light, whereas when they aggregate, their absorption spectrum redshifts into the NIR. Six—eight-week-old male mice were implanted with murine squamous cell carcinoma cells (SCCVII), and then 100 mg Au/mL (10% by weight) was injected into the tumor. The small animals were irradiated with a 820 nm laser at 1.5 W/cm^2^, reaching 48 °C for 40 s. After 3 min, the radiation therapy started with an X-rays dose rate of 7.5 Gy/min. The results obtained showed that in the presence of Au nanostructures, the radiation that is needed is less than 15 Gy. This was a good result considering that the conventional radiation dose necessary to control 50% of the tumor is 55.4 Gy. The experiment demonstrated the potential of clinically adopted AuNPs.

In recent years, AuNSs have also been gaining significant importance in the medical field thanks to their different properties. Their anisotropy, the presence of spikes around a spherical core, determines that they have a high surface/volume ratio, therefore bioconjugation is considerably more accessible [93]. Additionally, thanks to the presence of spikes, there is an enhancement of the local electromagnetic field at the tips. The latter part knocks out the oscillating surface electrons, transferring their energy to the atomic lattice and giving off heat at the metal–dielectric surface [104]. Since AuNSs possess a wide absorption cross section in the NIR range, they are suitable for PTT. Moreover, their ability to bioconjugate with dyes or antibodies causes considerable damage to the tumor cells irradiated with lasers at certain power densities [105].

The remarkable benefits induced by AuNSs in PTT were studied in vitro by Gao et al. [106] using 4T1 breast cancer cells. AuNSs, at concentrations of above 20 μg/mL, caused a great reduction of the cells’ viability (>90%) after the PTT treatment.

Li et al. [107] designed a nanoprobe of hollow, mesoporous silica (HMS) with AuNSs encapsulated with perfluorohexane (PFH) and surface modified with mPEG-SH (for short, HAPP) for a PTT treatment against glioma (C6 cancer cells) in vivo. The C6 tumor-bearing cells were divided in four groups: PBS control, PBS plus laser, HAPP and HAPP plus laser ones. The Au concentrations were considered to be in a range from 0.05 to 0.8 mM, and the mice were irradiated for 5 min under an 808 nm laser. The result showed that the tumor treated with HAPP died completely after two days, while the tumors treated without HAPP grew again after a few days.

The cancer treatment using heat can be induced by other several types of hyperthermia, including radiofrequency (RF)-based hyperthermia and/or modulated electro-hyperthermia (mEHT) [108,109]. Regarding RF-based hyperthermia, Thermotron RF-8 is the device that is commonly used. It produces dielectric heat by rapidly changing the radiofrequency by 8 MHz; mEHT on the other hand, focuses an electric energy field to the tumor area and generates heat on the membrane. 

Chen et al. [110] demonstrated the best efficiency in cancer treatments performed with mEHT comparing to the Thermotron RF-8 and hot water bath ones. They also analyzed the effect of the AuNPs and mEHT combination on cell killing. They synthesized different AuNP shapes (~50 nm), such as sphere-like AuNPs (SAuNPs), urchin-like AuNPs (UAuNPs) and rod-like AuNPs (RAuNPs). The human hepatocellular carcinoma cell line (Hep-G2) was used. The AuNPs influence was both investigated in the medium (extracellular AuNPs) and incorporated in the cells (cell-incorporated AuNPs). For the extracellular AuNPs evaluation, the Hep-G2 cells were divided in two groups and separately incubated with 25 ppm of SAuNPs, UAuNPs or RAuNPs. The first group was immediately placed in a hot water bath, while the second one suddenly received the mEHT treatment. For comparison, other two groups of Hep-G2 cells were treated with a hot water bath and with mEHT, but without AuNPs. The results showed that extracellular AuNPs did not modify the selectivity or the cell-killing effect of the mEHT method. For the cell-incorporated AuNPs evaluation, the two Hep-G2 groups were first incubated with AuNPs for 24 h, and then subjected to the two hyperthermia treatments. For the hot water bath treatment, the viability of the AuNP-incorporated cells ranged from 96% to 98%, while for the mEHT treatment, the viability ranged from 76% to 83%. It is much higher than those without cell-incorporated AuNPs (57%). In the last case, the AuNPs acted as protectors in the electro-hyperthermia cancer treatment. Moreover, this experiment revealed that the RF-induced heat generation of AuNPs was totally insignificant using the Thermotron RF-8. 

In the final analysis, thermal hyperthermia (MHT) also assumed a central role in the treatment of cancer diseases. It involves magnetic nanoparticles (MNPs) that, when they are subjected to an alternating magnetic field (AMF), cause a rise in temperature from 42 to 46 °C, contributing to the killing of the diseased cells. MHT can also be combined with other therapeutic strategies (radiotherapy, photo thermal therapy, photo dynamic therapy, etc.) to provide more efficient results in tumor treatments [111].

### 4.3. Photodynamic Therapy (PDT)

**Figure 9:**
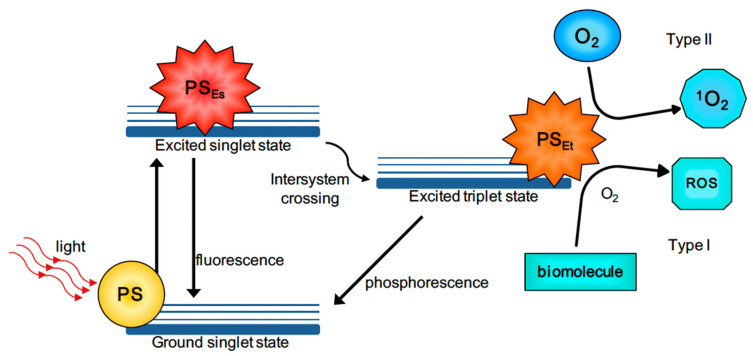
Reaction mechanism of PDT [113].

Photodynamic therapy (PDT) is a non-invasive cancer treatment that makes use of a photosensitizers (PS), molecular oxygen and light of different wavelengths depending on the biological tissue that needs to be treated. PS is a non-toxic dye which, when it is irradiated, induces the production of reactive oxygen species (ROS) and organic radicals [112] that kill cancer cells. In Figure 9, the detailed description of the mechanism of PDT is reported.

The PS, initially in the ground state, goes into an excited singlet state after the absorption of light. In this state, the PS has a very short lifetime (from nano- to picoseconds). The PS can either return to the ground state via fluorescence or heat emission, or it can switch to a triplet excited state through intersystem crossing. The PS in the triplet state has a slightly longer lifetime (from micro- to milliseconds) than the one in the singlet state. As in the previous situation, here too, the PS in the triplet state can either return to the ground state by means of the emission of phosphorescence. Alternatively, the photodynamic effect will occur. This means that there will be electron transfer between the triplet-state PS and the cell substrate (Type I reaction) or energy transfer between the triplet-state PS and molecular oxygen (Type II reaction). The type I reaction leads to the formation of ions and free radicals which, when they react with O_2_, generate oxygenated products (OH, H_2_O_2_ and O_2_^−^). On the other hand, the type II reaction will lead to the formation of highly reactive O_2_. ROS produced by the two reactions are responsible for the tumor cells’ death, which occurs by apoptosis, necrosis or autophagy [114].

In addition, PDT not only is responsible for eliminating the tumor mass, but it could prevent further formation over time thanks to the activation of the immune system. This happens because PDT generates acute inflammation, attracting immune cells. Some in vitro studies [115] have shown that by using a photo grid as a PS, PDT induces the fixation of complement 3 protein to the tumor cells. This is a signal that the innate immune system can recognize the same malignant cells in time and destroy them [116].

In order to make PDT efficient, the choice of the correct PS is fundamental. Usually, the light range used for PDT is from visible to NIR in order to cover deep tumors in different anatomical tissues. A high amount of ROS production following irradiation is crucial [117]. The choice of the PS must also consider other fundamental aspects for the success of the therapy. The PS should be easily synthesized, so that it can be produced in large quantities. Then, it is preferable that it is amphiphilic and photostable for it to be able to penetrate different types of tissues. Finally, it should have both a relatively long-life span in the triplet state and be soluble in water [118].

Although the PDT is one of the least harmful ones, as the damage to the surrounding healthy tissues is minimal, there are some aspects that could limit its efficiency. If cancer cells are in a hypoxic environment, a lack of oxygen can hinder the therapy. This is because the efficiency of the PDT depends on the amount of molecular oxygen present in the biological tissue. Furthermore, not all biological tissues involve the use of the same irradiation wavelength. In fact, just as water absorbs longer wavelengths, hemoglobin and melanin absorb smaller wavelengths. This results in an irradiation range from 600 to 1300 nm [119].

Another important aspect is the delivery of the PSs to the region of interest. Many of the PSs tested are hydrophobic, resultingly hindering the administration of PSs into the systemic circulation [120]. For all these reasons, the application of nanostructured materials, such as AuNPs, overcome this limitation.

Firstly, AuNPs are inert and biocompatible carriers for PS. PS dyes can be encapsulated or conjugated onto the NPs’ surface, providing a synergistic effect to the destruction of cancer cells after irradiation. Thanks to the AuNPs’ coating, the PSs are protected from immune cells that can destroy them during their circulation [121]. In fact, AuNPs are not recognized by the immune system, becoming a good barrier and an excellent delivery system for PSs [117].

The EPR effect enables a longer circulation of the AuNPs/PSs, thereby increasing the therapeutic effect [117,122].

Finally, the high surface area of AuNPs can be easily modified and functionalized with different ligands to improve PS absorption.

## 5. AuNPs for Applications in Cancer Imaging

The best cancer prevention tool is diagnosis. Today, there are several techniques to trace and recognize the disease in all its phases. 

To improve the diagnostic techniques, nanomaterials are applied. In many imaging methods, AuNPs act as excellent contrast agents, allowing researchers to make the distinction between tissues with similar absorption coefficients and cell tracing. 

Moreover, tumor mRNA-dependent drug carrier-based intracellular imaging is an influential technique used to control cancer progression. Qiao et al. [123] designed molecular beacon (MB)-functionalized AuNPs (AuNP-MB) that target intracellular cyclin D1 mRNA (a tumor mRNA of breast cancer). Fluorescent Doxorubicin (Dox), a drug used for many cancer treatments, was intercalated in the double-stranded stem region of MB (AunP-MB(Dox). In this way, MB carried Dox, quenched the fluorescence and, once the MB loop hybridized with DNA/RNA target, it opened and releases Dox. They incubated three cell types with AunP-MB(Dox): SK-BR-3 cells treated with cartilage polysaccharide that down-regulates D1 mRNA expression, untreated SK-BR-3 and MCF-10A. The results obtained show a correlation between the intensity of fluorescence with the tumor mRNA amount in the viable cells. The fluorescence was greater in the cytoplasm and in the nuclei, and finally, the red fluorescence of the untreated cells was much higher than it was in polysaccharide-treated cancer cells. 

Among the different diagnostic techniques, computed tomography (CT), photoacoustic imaging (PAI) and single-photon emission computed tomography (SPECT) were applied in combination with AuNPs. 

### 5.1. Computed Tomography (CT)

Computed tomography, which was developed by Wilhelm Röntgen in 1895, allows researchers to acquire three-dimensional images of a specific anatomical area to which the tumor is confined to identify the lesions in a precise manner. The three-dimensionality is achieved by obtaining sequential axial images resulting from the attenuation of a large number of X-ray transmissions through the patient’s body [124].

The attenuation process involves the exponential decrease in the X-ray intensity due to the variation in energy, number and direction of the incident beam. This phenomenon is mainly due to two radiation–matter interactions: the photoelectric effect and Compton scattering [125]. These two phenomena are not the only interactions that occur once the beam meets the biological tissue but observing them is more suitable for the clinical aims.

The photoelectric effect involves electrons bound to the nucleus. If the incident X beam has a greater energy than that of an electron in an innermost shell (usually the K or L shells), the latter is emitted (photoelectron), and the photon is absorbed. The vacancy created by the ejected electron is filled by electrons from the outermost shells, causing the release of secondary radiation. The radiation released, together with the following emission of the photoelectron, are responsible for the damage to the surrounding tissues. The energy threshold required to eject an electron is called the absorption edge (K-edge), and it takes on different values depending on the contrast agents used in CT. It is 33.2 KeV for iodine, 34.7 KeV for barium and 80.7 KeV for Au.

In Compton scattering, the photon interacts with a free electron, inducing its ejection. The photon is consequently scattered, helping to decrease the image contrast [126].

The equation that describes the radiation attenuation process is the Lambert–Beer law (Equation (1)):

(1) $$I\left(x\right)=I_{0}e^{-µx}$$

in which *I*_0_ is the intensity of the incident beam, *I* is the intensity after the interaction with matter, *x* is the thickness of the crossed tissue and µ is the attenuation coefficient. The attenuation coefficient µ is the sum of the respective absorption coefficients relating to the photoelectric effect (µ_pe_) and Compton scattering (µ_Compt_) (Equation (2)):

(2) $$µ={µ}_{pe}+{µ}_{Compt}$$

Thanks to the “filtered back projection” method, each attenuation value of each beam is rear projected along its trajectory. Each numerical value of the set obtained corresponds to a point in the image reconstruction field. The image is reconstructed by transforming these values into grayscale using the Hounsfield units (Equation (3)):

(3) $$HU=k\frac{{µ}_{t}-{µ}_{w}}{{µ}_{w}}$$

in which µ_t_ and µ_w_ are, respectively, the attenuation coefficient of the tissue and that of water (it is considered as a reference value), while k is usually equal to 1000.

Tissues with a density that is greater than the density of water have HU numbers that are bigger than 0, otherwise negative HU values are obtained. In current CT, the Hounsfield units’ range is from −1024 HU to +3071 HU. In the CT image, lots of attenuation is represented by white, while a minor degree of attenuation is represented by black.

However, when small tumors (dimensions of below one centimeter) or tissues with low contrast are considered, good images, from which the various components can be distinguished, are obtained using CT. In these cases, a so-called contrast agent (CA) is used. The contrast agent has a different density (lower or higher) than that of the surrounding tissue and/or anatomical area [127].

The photoelectric effect occurs when the energy of the photon beam is almost equal to the binding energy of the electrons. Since that the attenuation coefficient is inversely pro-portional to the energy of the photon beam ($\mu_{\mathrm{pe}}\propto \frac{1}{E^{3}}$), if the energy of the beam increases even more, less absorption will occur [128,129]. Compton scattering, on the other hand, is more likely to occur for higher energy photons. Then, if the energy of the beam increases, the photons will be able to pass through the body. However, just as a low-energy beam becomes extremely harmful to the patient due to the high dose of radiation, a high-energy beam significantly degrades the image quality. A fair compromise is therefore necessary in the medical field. 

The mass attenuation coefficient is greater as the atomic number Z increases, leading to better image contrast. In essence, the contrast agent must have a high atomic number Z to obtain excellent contrasts, and a lower dose of radiation is used [130]. Nowadays, the most used contrast media in CT are iodinated derivatives. Thanks to their relatively high atomic number (Z = 53) and their electron density of 4.9 g/cm^3^, they exhibit a good attenuation coefficient [130]. Iodinated derivatives are water soluble, non-toxic, and they have a low molecular weight. However, there are some characteristics that make iodine-based contrast agents not always suitable for CT, for example, a low K-edge energy, which does not help to improve the image contrast. In addition, the iodine-based contrast agents have a short blood circulation time, and they are not recommended for people with hypersensitivity to iodine. Moreover, when iodine-derivatives are used in vivo, they undergo rapid renal excretion [129].

Although iodinated contrast agents are an integral part of CT, they have side effects, such as life-threatening contrast-induced nephropathy (CIN). CIN implies renal dysfunction after the use of a contrast medium [131]. 

So, the use of AuNPs as a contrast agent can improve the imaging and overcome several limitations of the traditional compounds that are described above. AuNPs have an atomic number (Z = 79) that is greater than that of iodine and a high electron density (19.32 g/cm^3^). Moreover, they are non-toxic and biocompatible in vivo [130]. This property is necessary for a good contrast agent, since a lot of them are toxic and can have collateral effects on patients. The most useful advantages to using AuNPs as a contrast agent are their long vascular half-life and their combination with different imaging techniques [132].

One of the first studies on AuNPs as a contrast agent for X-ray imaging was reported by Hainfeld et al. [78]. AuNPs with a size of 9 nm were administrated by intravenous injection into mice, and the authors found that X-ray imaging could detect such particles and their biodistribution within the animal’s body [133]. In addition, this new contrast tool is not toxic and is easily excreted by the kidneys.

In addition to using spherical AuNPs, AuNSs have also been studied in diagnostic imaging due to their unique properties. In particular, thanks to their strong absorption, they are tested as a contrast agent to improve the contrast of images [134]. Furthermore, the AuNSs passively accumulate in the tumor area due to the EPR effect. The EPR effect exploits the possibility of tuning the size of the NPs, which is preferably below 100 nm. Instead, the lymphatic system, under normal conditions, should bring the AuNSs back into circulation. This does not happen due to the presence of the neoplasm [135].

AuNSs have been demonstrated to be highly efficient. The standard treatment for brain glioma is surgery. In many cases, the results are not satisfactory, leading to the formation of further malignant sites or metastases. This is probably due to the blood–brain barrier (BBB), which prevents external agents from crossing the blood parenchyma [136].

### 5.2. Photoacoustic Imaging (PAI)

Photoacoustic imaging (PAI) is a tool of considerable interest in the biomedical field for the diagnosis of cancer diseases. It is based on the photoacoustic (PA) effect. It consists of the irradiation of the biological tissue under examination by means of nanosecond pulsed laser light (pulse duration < 10 ns) in the NIR range. When they are in the target tissue, the chromophores absorb the laser pulses and convert them into heat. The result is a rise in temperature, which is responsible for thermoelastic expansion. Acoustic waves are generated and detected using special transducers, allowing the formation of the final image [137]. In PAI, the wavelengths used are in the range of 650–950 nm (NIR-I) and 1000–1400 nm (NIR-II); in these wavelength windows, the tissues are transparent, reaching a good penetration depth of several centimeters [138].

In PAI, non-ionizing radiation is used, thus avoiding consequent long-term harmful effects; in addition, PAI has high sensitivity and high spatial resolution (down to several microns) with a penetration depths of up to 5–6 cm, while the scattering phenomena between the detected signal and biological tissues are minimal. However, the road to the effective use of PAI could still be long given the current experimentation of this imaging method [139].

PAI can exploit endogenous contrast agents such as hemoglobin, lipids, water and others. These chromophores have their own unique absorption spectrum, which can sometimes resemble that of another chromophore. For this reason, exogenous contrast agents are often used [140].

AuNPs are excellent exogenous agents. Thanks to the phenomenon of surface plasmon resonance (SPR), the AuNPs absorb the incoming light with consequentially high absorption peak in the UV-vis-NIR spectrum. In addition to the principal physicochemical properties described in the previous sections, AuNPs also exhibit low thermal diffusivity, an important feature for photoacoustic diagnosis [141]. Au nanorods are among the most widely used NPs as an exogenous contrast agent in the experimental phase.

**Figure 10:**
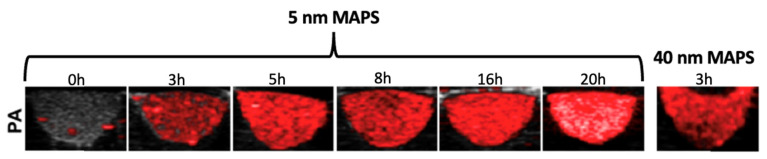
PA images of EGFR+ A431 cells labeled with 5 nm MAPS for different periods of time. PA images of 40 nm MAPS for comparison [142].

Han et al. [142] synthesized antibody-targeted AuNPs of different sizes to evaluate their efficacy during PAI. There are two problems related to the size of the NPs: NPs with a diameter greater than 20 nm would not be subject to the clearance process, but they could cause harmful effects for humans if they are kept in the body for excessively long times. Instead, smaller diameter NPs could be more easily expelled due to clearance. They referred to their EGFR-targeted 40 nm and 5 nm AuNPs as “molecularly activated plasmonic nanosensors” (MAPS). Then, they compared them in order to evaluate their ability to generate an effective and strong PA signal from labeled cancer cells. The comparison between 5 nm and 40 nm EGFR-targeted AuNPs during the PAI of squamous carcinoma cells (A431) showed the same signal intensity after 3 or 6 h of incubation (Figure 10). 

No signal was observed with the bare AuNPs. Despite both the 5 nm and 40 nm signals being almost equal, the 40 nm MAPS-labeled cells showed scattering phenomena. This characteristic makes 5 nm MAPS more promising than the 40 nm MAPS as an exogenous agent. 

**Figure 11:**
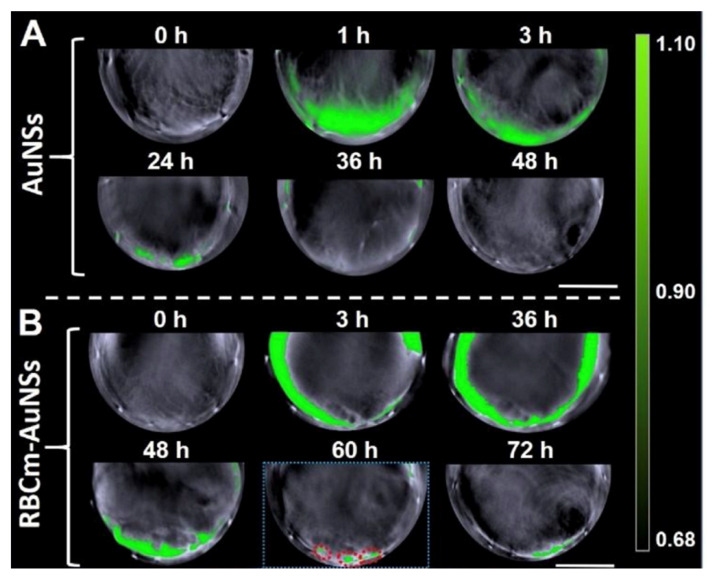
PA images of the two groups of mice after injection of (**A**) AuNSs and (**B**) RBCm-AuNSs. The images cover different periods of time from 0 h until 48 h [143].

AuNSs are also applied in the PAI technique. Huang et al. [143] used AuNSs for their tunability of the NIR spectrum and the presence of spikes which act as “antennas”, thereby improving the optical absorption. They designed an RBC membrane-coated AuNS probe (RBCm-AuNS) to overcome the mononuclear phagocytic systems (MPS) clearance and accumulate at the liver tumor site. Six male mice with an Hep-G2 tumor at the liver site were divided into two groups: one group was injected intravenously with 200 µL of RBCm-AuNS, and the other one was injected with the same amount of bare AuNSs (Figure 11).

Using the AuNSs, the PA signal was evident until the third hour, but after this time it decreased, while when the researchers were using the RBCm-AuNS instead, the PA signal remained clearly visible for the entire duration of the treatment. Furthermore, at 60 h, the imaging revealed three small hepatocellular carcinomas with dimensions of less than two millimeters. RBCm-AuNS are suitable to be an efficient probe for PAI, ensuring its own non-expulsion, and therefore, accumulation at the tumor site.

Neuschmelting et al. [144] synthesized AuNSs/SiO_2_ for PA image-guided surgery. The result was excellent thanks to the high spatial resolution (of the order of a sub-millimeter) and excellent photostability. Furthermore, due to the depth of penetration and the ability to accumulate directly at the tumor site of the mouse brain, the PA images were characterized by high sensitivity and an excellent SNR [145].

### 5.3. Single-Photon Emission Computed Tomography (SPECT)

Single-photon emission computed tomography (SPECT) is a diagnostic technique that is useful for the diagnosis of patients. It provides the intravenous administration of radiolabeled drugs called radiopharmaceuticals in order to monitor the tumor progress. The operation of SPECT is based on the detection of γ-photons emitted by radionuclides and detected by an array of γ-cameras positioned externally to the patient. The energy of the detected photons varies from 100 to 250 keV [146]. Usually, the drugs are labeled with ^99m^Tc, ^111^In, ^123^I e and ^201^Tl. Furthermore, more efficient imaging is often achieved through a combination of CT/SPECT [147].

Although the use of SPECT alone can present both a low spatial resolution and a remarkable penetration, the CT/SPECT combination shows a high sensitivity and an improvement of the spatial resolution. Thus, it is possible to detect functional biological abnormalities more efficiently [148].

Regarding the brain imaging, the use of SPECT compared to other instruments in nuclear medicine (such as PET) has non-negligible advantages. These include the low radiation dose when ^99m^Tc-based radiopharmaceuticals are used (which is especially important for pediatric patients). The use of SPECT alone without CT also involves less radiation exposure. Furthermore, SPECT enables dual isotope imaging thanks to the innovative cameras on the market [149].

Different AuNP shapes have been labeled with radionuclides to study their diagnostic efficiency in SPECT [150].

A single NP can bind to multiple radionuclides, which is contrary to the most common radiopharmaceuticals. In addition, they can reach and stand in the tumor mass (EPR effect) despite the spaces between the endothelial cells of the weak blood vessels due to their small size [151].

**Figure 12:**
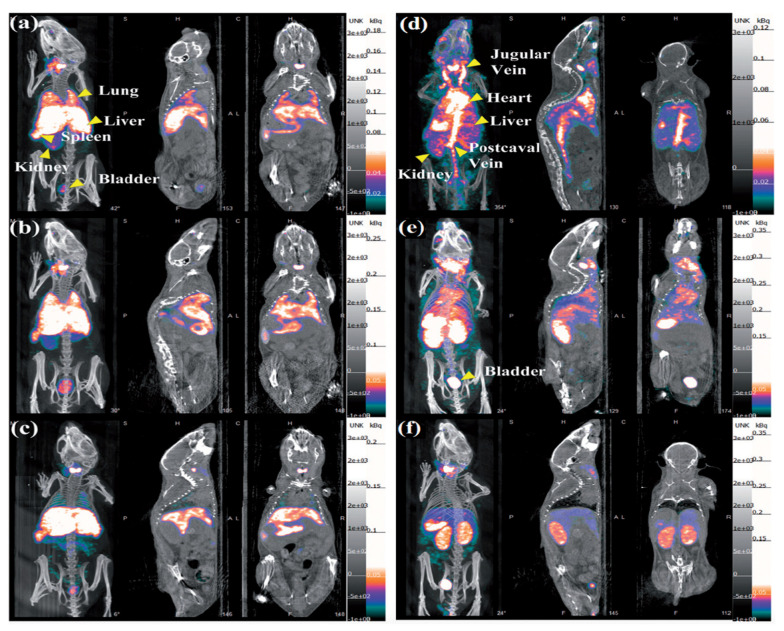
SPECT/CT images of mice injected with ^99m^Tc-Au-Ac-PENPs at (**a**) 0.5 h, (**b**) 1 h and (**c**) 2 h; SPECT/CT images of mice injected with ^99m^Tc-Au-Gly-PENPs at (**d**) 0.5 h, (**e**) 1 h and (**f**) 2 h [152].

L. Zhao et al. [152] used the ^99m^Tc radioisotope for their experiment due to the low energy of γ-rays (140 keV), its half-life and its low cost. They synthesized ^99m^Tc-labelled Au PENPs (^99m^Tc-Au-PENPs) with two different surface groups (^99m^Tc-Au-Ac-PENPs and ^99m^Tc-Au-Gly-PENPs, respectively). Nude mice were injected with both the labelled agents, and then imaged with SPECT/CT (Figure 12).

As shown in Figure 12, it is clear that ^99m^Tc-Au-Ac-PENPs accumulated in the lungs and kidneys 0.5 h after the injection (Figure 12a). The maximum accumulation was recorded 1 h after the injection (Figure 12b). After 2 h, they had accumulated in the liver and spleen (Figure 12c). On the other hand, ^99m^Tc-Au-Gly-PENPs accumulated in the blood 0.5 h after the injection, with a weak signal in the liver (Figure 12d). Then, they accumulated in the kidneys and bladder (Figure 12e), and then, they cleared (Figure 12f). With from this evidence, it has been demonstrated that Au PENPs are an optimal contrast agent for SPECT/CT imaging in vivo, with high biocompatibility and biosafety.

J. Zhu et al. [153] developed charge conversional Au PNPs with ^131^I-labeling for improving SPECT/CT imaging. They considered polyethylenimine (PEI) because it can entrap AuNPs in the cavity. Then, they integrated ^131^I and AuNPs in one PEI nanosystem in order to achieve a very high efficiency in the SPECT/CT imaging. The final ^131^I-APAS-Au PNPs were generated and used in their experiment. C6-tumor-bearing mice were divided in two groups: first group was injected intravenously with ^131^I-APAS-Au PNPs (100 µL; 400 µCi), and the second one was injected with ^131^I-Au PNPs (100 µL; 400 µCi). The mice were imaged with the SPECT system at different time points after injection. For the CT images, they used two other groups of mice that were injected, respectively, with APAS-Au PNPs (0.1 M; 100 µL) and Au PNPs (0.1 M; 100 µL). They obtained similar results for both the CT and SPECT images. In the first case, 4 h and 6 h after the AuNPs administration, the brightness of the mice tumor injected with APAS-Au PNPs was almost two times higher than the one of the mice injected with Au PNPs. In the second case, the SPECT signal of the mice treated with ^131^I-APAS-Au PNPs was much higher at every time point than those of the mice treated with ^131^I-Au PNPs.

**Figure 13:**
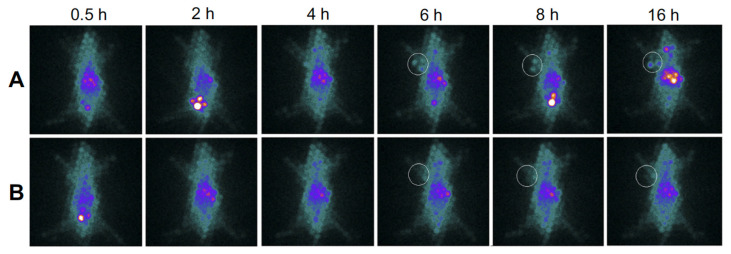
SPECT images of mice treated with (**A**) BmK CT-Au PENPs-^131^I and (**B**) Au PENPs, respectively, at different time points post-injection. The tumor site is highlighted by white circle [154].

In another experiment, Sun et al. [154] always used PEI, generating ^131^I-labeled Au PENPs modified with the glioma-targeting peptide BmK CT (BmK CT-Au PENPs-^131^I) for targeted SPECT/CT. The glioma-bearing nude mice were divided into two groups and injected intravenously with BmK CT-Au PENPs-^131^I and with Au PENPs, respectively. The SPECT signal in the mice injected with BmK CT-Au PENPs-^131^I increased considerably 2 h post-injection, and it reached a peak at 8 h post-injection (Figure 13A). In reverse, for tumors treated with Au PENPs, a weak signal was visible only after 8 h post-injection, without any other tumor uptake (Figure 13B).

Similar results were obtained from the mice imaged using the CT system.

From numerous experiments that have been carried out, it is clear that the use of radiolabeled AuNPs is a tool that significantly increases the diagnostic efficacy of SPECT, also guaranteeing high radio stability and biocompatibility in vivo. Moreover, it allows the correct combination of SPECT/CT systems to achieve both a good diagnostic sensitivity and a greater depth of penetration into the tumor tissues.

## 6. Functionalized AuNPs for Cancer Therapy 

The broad study of AuNPs in cancer therapy, both for imaging and tumor treatments, involves not only bare AuNPs, but also different AuNP shapes functionalized with different ligands and/or functional compounds. 

Concerning the functionalized AuNPs used in RT, Zhang et al. [155] studied the radio sensitization effect of 4.8, 12.1, 27.3 and 46.6 nm AuNPs coated with PEG in vitro and in vivo. For the in vitro study, they treated HeLa cells with PBS as a control sample, and then, they exposed the cells to functionalized AuNPs at the concentrations of 0.05 and 0.1 mM for 24 h. After, the cells were irradiated with 0, 2, 4, 6 and 8 Gy of ionizing radiation. The cells treated with 0.05 mM of AuNPs and 12.1 nm PEG-coated AuNPs performed the strongest radiation enhancement. The group incubated with 0.1 mM of AuNPs and 12.1 and 27.3 PEG-coated AuNPs caused the higher growth inhibition after 8 Gy RT irradiation. For the in vivo study, U14 tumor models were generated by the subcutaneous injection of cancer cells into female BALB/c mice. One hundred µL of 4.8, 12.1, 27.3 and 46.6 nm PEG-coated AuNPs were injected into the mice, and after this, they were irradiated by 5 Gy gamma-rays. The injection of 12.1 nm functionalized AuNPs showed the highest radiation enhancement effect, with an appreciable decrease in the tumor volume, while the 46.6 nm functionalized AuNPs showed disappointing results. Regarding the study of tumor treatment in PTT, Majidi et al. [156] tested the efficiency of Au nanoshells against melanoma cancer cells (A375). In detail, they synthesized folic acid (FA) conjugated with silica Au core–shell NPs (FA-SiO_2_@AuNPs). Under NIR laser irradiation (808 nm, 4 min of irradiation time exposure), the AuNPs reduced the cancer cells viability by about 48%.

In another work aptamer-conjugated AuNSs (Apt-AuNSs) were synthetized for a PTT treatment [157]. HeLa cells were incubated for 2 h with 150 µg/mL of Apt-AuNSs (size 80 nm), and then irradiated for 10 min under 808 nm laser. After the treatment, the cell viability decreased from 86.29% to 13.5% when the laser power density ranged from 0.5 to 2.0 W/cm^2^.

Dixit et al. [158] studied AuNPs that were complexed with peptides, which acted as nanocarriers of the drug sensitizers phthalocyanine 4 (Pc4) in PDT. The tumor was a polymorphic glioblastoma. The obtained results showed that targeted AuNPs were six times more efficient than the non-targeted AuNPs were.

The LSPR effect is also relevant in therapy. It can cause hyperthermia, improving the PDT effect [159].

Wang et al. [160] functionalized Au nanorods (AuNRs) for the PDT of the leukemia cell line using an aptamer switch probe (ASP) linking the PS chlorin e6 (Ce6) on the surface of the AuNRs. The new nanoplatform AuNR-ASP-Ce6 demonstrated a better PDT treatment than PDT alone did, and it was characterized by specificity, high efficiency, and the improved ability of the Au nanorods to carry many PSs. In another work, the AuNRs, which were obtained by using seed and growth technique, showed an aspect ratio of 3.8 (length: 35 nm; width: 9.3 nm) [161]. Then, they were conjugated with a hydrophilic and anionic PS, indocyanine green (ICG) after the functionalization of PSMA polymers on nanorods’ surface. The complex AuNRs-PSMA-ICG was used for a PDT treatment of A549. The singlet oxygen quantum yields of ICG and Au-PSMA-ICG were measured against a value reference. They turned out to be 0.112 for ICG and 0.160 for Au-PSMA-ICG. This result demonstrates that the amount of singlet oxygen was higher for AuNRs-PSMA-ICG, thus improving the efficiency of the PDT treatment.

Wang et al. [162] functionalized AuNSs by Chlorin e6 (GNS-PEG-Ce6) to improve the effect of the PDT on breast and lung cancers. The fluorescence of Ce6 was reduced once that the PS anchored on the GNS-PEG’s surfaces due to the plasmonic effect. Moreover, after laser irradiation at 671 nm, the ^1^O_2_ quantity produced by GNS-PEG-Ce6 was very high (about 75% of free Ce6), thereby improving the PDT efficiency in a remarkable manner.

For cancer imaging methods, Chen et al. [163] demonstrated that targeted AuNPs functionalized with Trastuzumab improved CT breast tumor imaging. In particular, the AuNPs conjugate was applied to image HER-2 positive tumors in vivo. For the experiments, they used the Generation 5 PAMAM dendrimer conjugated with AuNPs (G5-AuNP), DOTA-Gd and Trastuzumab (G5-AuNP-Gd-Trastuzumab). For comparison, G5-AuNPs-Gd were used as contrast agent as well. Immunodeficient BALB/c nude mice with A549 cells were injected with 100 µL of G5-AuNP-Gd-Trastuzumab for an in vivo CT (80 kV, 5 mA). The mouse images were acquired by CT both pre-injection and at 1, 4, 8 and 12 h post-injection. As early as 4 h after the first scan was performed, the CT signal intensity increased by 13% compared to that of G5-AuNP-Gd, as well as the intensities of the scans taken after 8 h.

In an in vivo study [164], AuNSs (80 nm) were modified by PEGylation in order to reduce the systemic clearance in a cerebral micro angioma treatment. While the conventional contrast agent demonstrated intravascular intensity for less than 30 min, the AuNSs were stable for many hours without extravasation. Furthermore, the AuNSs accumulated more in the tumor area rather than in the surrounding tissues, minimally penetrating the BBB.

Sun et al. [165] synthesized glycol chitosan-coated AuNPs (GC-AuNPs) and analyzed their accumulation in the tumor site through CT. They used CT-26 colon cancer cells that were injected into mice. The NPs accumulated successfully in the tumor site, acting as contrast agent. However, it is important to select the most suitable polymer for the NPs functionalization because polymers also play an important role in contrast agent activity.

In another in vivo CT imaging study [166], folic acid (FA) AuNPs bound by cysteamine (Cys) were used to target human nasopharyngeal head and neck cancer. Targeted FA-Cys-AuNPs and non-targeted AuNPs with a spherical shape and mean diameter of 15 nm were injected intravenously into different mice. Then, CT scans were performed at different time points. In both scans, the contrast of the tumor image is clearly better. Targeted AuNPs can attenuate X-ray intensity, while non-targeted AuNPs cannot. Moreover, from 3 to 6h after the injection, the intensity of X-radiation decreased in the tumor.

**Figure 14:**
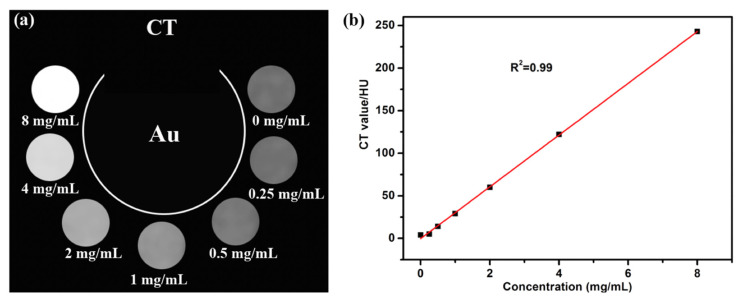
(**a**) CT images for different GNS-FA concentration; (**b**) Hounsfield values dependent on the AuNSs concentrations. Reprinted (adapted) with permission from [167], Copyright 2021 American Chemical Society.

In a recent experimental work, [167] the authors demonstrated the further efficiency of AuNSs as a contrast agent in CT using the HeLa cell lines. Tumor-targeting polymer folic acid-terminated polyethylene glycol thiol-modified AuNSs (GNS-FA) were obtained, and the authors evaluated their potential application in CT imaging. When the concentration of AuNSs increased, the brightness proportionally increased. In Figure 14, we report (a) the in vitro CT images using different concentrations of functionalized AuNSs and (b) the Hounsfield values, which are dependent on the AuNSs concentration.

AuNSs appear to be a more promising and applicable tool in CT for image contrast improvement [167] than other Au-based nanostructure shapes.

**Figure 15:**
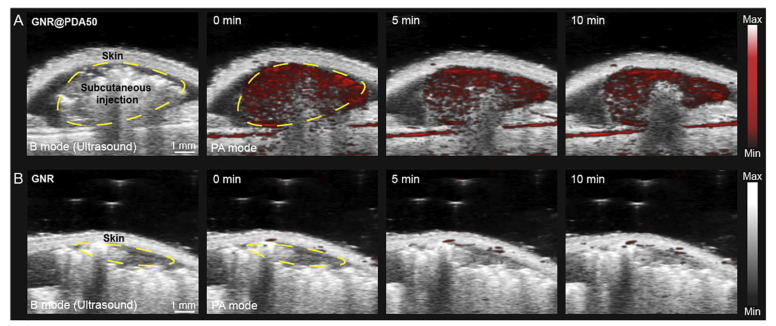
(**A**) PA images with Au nanorods@PDA as exogenous contrast; (**B**) PA images with Au nanorods as exogenous contrast. Reprinted (adapted) with permission from [168]. Copyright 2021 American Chemical Society.

Concerning the PAI, Yim et al. [168] used Au nanorods as exogenous contrast agents because of ability to absorb in the NIR-II range due to their aspect ratio. However, for their thermodynamic instability, these nano-objects can undergo deformation phenomena following laser irradiation. For this reason, the authors reported the organic molecule-mediated formation of GNR-melanin hybrids (Au nanorods@PDA), generating a protective layer of polydopamine (PDA) on the nanorods. The stability of these new NPs in vivo as an exogenous contrast during PAI was showed, and they were compared with conventional Au nanorods without surface modifications. Three mice were injected and imaged 10 min later. Figure 15 shows the significant difference in contrast between the PAI images, which were acquired, respectively, using Au nanorods@PDA and the conventional Au nanorods.

It is evident that Au nanorods@PDA have a higher PA intensity and signal stability than Au nanorods do. In detail, the PA intensity of Au nanorods@PDA is 10-fold higher than that of the Au nanorods, and it has been maintained even after 10 min after irradiation. After the same time, instead, the PA intensity of Au nanorods disappeared.

Wang et al. [138] achieved AuNRs modified with thiolated poly(ethylene glycol) (thiol-PEG) (NRs@PEG) and used them for in vivo multifunctional PAI. NRs@PEG have absorption bands in the range of NIR-I (from 650 to 110 nm). A mouse was intravenously injected with AuNR@PEG (and with commercial AuNPs and AuNRs for comparison). The cerebral blood vessels of the mouse were imaged before and after the injection. The wavelengths were tuned to 828, 680 and 532 nm. The results demonstrated that upon irradiation at 532 nm, NRs@PEG had a signal-to-noise ratio (SNR) that was like the one of the commercial AuNPs; instead, upon irradiation at 680 and 828 nm, NRs@PEG provided the highest SNR (1.1–1.5 times higher). The PEG shell helped the biocompatibility improvement of AuNRs, revealing a low toxicity rate.

Umehara et al. [169] modified the AuNRs with 2-nitroimidazole derivatives (NIs) (G-NIs) to detect tumor hypoxia in vivo by PAI. They used colon 26 cancer cells (CT26) that were transplanted into mice and injected them intravenously with G-NIs. By comparing both the effects of G-NIs and G-PEGs on the PA images, the former accumulated in the hypoxic tumor environment, while the latter only contributed to generating an unclear image. They concluded that G-NIs are a promising contrast agent for the visualization of tumor hypoxia by PAI.

F. Chen et al. [170] first achieved PEGylated AuNPs by synthesizing AuNPs capped with polyethyene glicol-5000 (PEG-5000) [171] for a SPECT analysis, thus, they conjugated the PEGylated AuNPs with dimethyl-maleic amide, making them pH-responsive AuNPs (dAuNPs). Then, using dAuNPs and Na ^131^I, they obtained ^131^I-dAuNPs with a spherical shape. Tumor-bearing nude mice were divided into three different groups. The first group was injected intravenously with conventional AuNPs, the second was injected with dAuNPs, and the third one was injected with radiolabeled probes (^131^I-dAuNPs) (100 µg/mL). The first two groups were imaged with PAI, and the third one was imaged with SPECT. The SPECT images revealed a strong diagnostic potential. Eight h after the injection, the tumor zone demonstrated a strong absorption of the NPs, reaching the maximum absorption between 12 and 16 h post-injection.

## 7. AuNPs Toxicity

The AuNPs studies described in literature demonstrate their applications in the nanomedicine field to promote and improve cancer treatment efficiency [172]. Nevertheless, there is no lack of experimentation showing, under certain conditions, the possible AuNPs toxicity. 

AuNPs, as such, are not toxic, however, in a solution, they may acquire a degree of toxicity due to some capping agents and/or chemical residues [173].

Toxicity also depends on other factors such as the AuNPs’ concentration, dose, size and shape [174]. Indeed, smaller NPs (from 2 to 20 nm) seem to be more toxic because of their ability to accumulate more easily in the liver and spleen due to their good circulation time and biodistribution in comparison with those of larger NPs [175]. 

Isoda et al. [176] investigated the toxicity of 10, 50 and 100 nm diameter AuNPs (GnP10, GnP50 and GnP100) in mice and examined the effect of the NPs on the toxicity of paraquat (PQ, a liver-kidney toxin), cisplatin (CDDP) and 5-aminosalicyclic acid (5-ASA, a common anti-inflammatory). For comparison, they analyzed the exposure to 4 mg/kg of AuNPs. No adverse effects on the kidneys, liver, heart, lung and spleen were found. This study demonstrated that the co-administration of GnP10 or GnP50 and CDDP increased alanine transaminase (ALT) and induced liver damage; the co-administration of GnP10 or GnP50 and 5-ASA caused liver and kidney damage; the co-administration of GnP10 and PQ increased the blood urea nitrogen (BUN) and induced renal damage, but not liver damage. The larger particles of GnP100 did not cause any adverse effects. This shows how smaller NPs are more dangerous than bigger ones.

Steckiewicz et al. [177] investigated the cytotoxicity of AuNPs rods (≈39 m length), AuNSs (≈215 nm tip-to-tip length) and AuNPs spheres (≈6.3 nm) against human fetal osteoblast (hFOB 1.19), osteosarcoma (143B, MG63) and pancreatic duct cell (hTERT-HPNE) lines. They measured the influence of AuNPs on the levels of proapoptotic protein (Bax) and anti-apoptotic proteins (Bcl-2). They found that the AuNPs spheres did not decrease the viability of hFOB 1.19 and MG-63 cells. Moreover, using 5 µg/mL of NPs concentration, the viability of 143B cells decreased, but the effect was lower than those of other shapes. Both AuNSs and AuNRs caused the increase in Bax and decrease in Bcl-2 protein expressions. Furthermore, the AuNPs penetrated through the cell membrane, causing ultrastructural changes. Despite this, AuNSs are the most cytotoxic ones against human cells. 

Zhang et al. [178] studied the in vivo toxicity of 5, 10, 30 and 60 nm PEG-coated AuNPs. The 5 and 10 nm AuNPs accumulated in the liver, the 30 nm AuNPs accumulated in the spleen, and the 60 nm AuNPs also accumulated in other organs. They concluded that the 10 nm and 60 nm AuNPs exhibited a higher toxicity than the 5 nm and 30 nm AuNPs did [179].

Another aspect that affects the toxicity of AuNPs are the synthesis methods. The green chemical procedures can be an alternative to reduce the use of toxic solvents and reagents. Moreover, the type of functionalization and the high rate of AuNPs accumulation in the spleen and liver contribute to the AuNPs toxicity rate [180].

Das et al. [181] studied the toxicity of three water soluble spherical 20 nm diameter-AuNPs, whose surfaces were modified with aspartic acid or with trisodium citrate dihydrate or with bovine serum albumin. Both male and nulliparous female mice were given 0.5 mL of AuNP suspension orally. The AuNPs modified with bovine serum albumin did not show any deleterious damage to the liver histology. The AuNPs and AuNPs modified with aspartic acid instead caused serious damage to the liver, such as hepatocytic disarray, the infiltration of inflammatory cells in the portal triad dilatation of the sinusoids and a congested central vein. The tissue of the AuNPs modified with trisodium citrate dihydrate treated mice exhibited some tubular alterations. On the contrary, all of the AuNPs had no toxic effect on the mouse hearts, spleen, lungs and testes. These results showed that there was a correlation between capping materials and the toxicity of AuNPs in vivo.

## 8. AuNPs Clinical Trials and Scientific Skepticism

Albeit, while several experimental research studies have focused on AuNPs-based cancer therapies, no Au platform has still clinically approved for the treatment of human cancer in vivo. One limitation regards their accumulation in organs such as the liver and spleen, although a large part is still excreted [182,183]. 

However, as reported by the NIH library database (Clinicaaltrials.gov), there are some ongoing clinical trials regarding AuNPs-based therapies. 

The first clinical trial (identifier: NCT00356980), which begun in 2006, involved Aurimune CYT-6091 (Cytimmune), the first tumor-targeted nanostructure consisting of 27 nm pegylated AuNPs conjugated with a tumor necrosis factor alpha (TNF). CYT-6091 (50 to 600 µg/m^2^) was injected into patients with solid tumors. The end of phase I demonstrated that the administration had no side effects and that CYT-6091 was well tolerated at doses that were greater than maximum tolerated dose for native TNF [184]. 

NU-0129 is a spherical nucleic acid (SNA) AuNP targeting the BCL2L12 oncoprotein in glioblastoma multiforme or gliosarcoma. In SNA, RNA is densely packed on the AuNP’s surface, leading to a stable nanostructure. In phase 0 of this clinic trial (identifier: NCT03020017), NU-0129 was intravenously injected into patients at an SiRNA dose of 0.04 mg/kg, 20 to 28 h prior to surgery. The SNA was able to cross the BBB and to suppress oncoprotein expression. This study reveal a reduction of the tumor-associated protein BCL2L12 without collateral effects being induced [182,185].

AuroShell, a silica–Au core–shell NPs, is being used in clinical trials for thermal ablation in patients with lung tumors (identifier: NCT01679470) and head and neck tumors (identifier: NCT00848042) [186].

However, despite the breakthroughs that have been made and are ongoing in the nanomedicine field, there is some skepticism regarding the anti-cancer medicine that follows three fundamental criteria. The latter one is established in order to create suitable nanostructures for cancer therapies, and they include: (1) nanomedicines must objectively increase their own accumulation in the tumor area due to the EPR effect; (2) the systemic circulation of the nanomaterials must be significantly prolonged due to surface modifications of the NPs; (3) a universal nano delivery platform based on both the EPR effect and long systemic circulation must be developed so that different anticancer drugs can be delivered [187]. These three basic pillars of nanomedicine have raised quite a few questions among skeptics. These include doubts about (1) the real existence of tumor EPR with respect to normal tissues; (2) the EPR enhancement action by nanomaterials with respect to free drugs; (3) the real usefulness of the long circulation time of nanomedicines, which occurs only if they can further improve the EPR effect compared with that of the free drugs; (4) the possibility of improving the drugs’ efficacy through a unique nano delivery platform if the principles of EPR and long circulation are invalid in human cancers [188]. 

Both the interest in these nanoplatforms and skepticism from researchers necessitate a better understanding of nanotechnology in medicine so as to improve its efficiency in therapeutics. The many doubts about the feasibility of the use of nanostructures for a cancer treatment should serve as a springboard to further investigate the properties and therapeutic utility of NPs in cancer patients.

## 9. Discussion and Conclusions

In this review, we have highlighted the properties of AuNPs, focusing the attention on the advantages to their use in imaging and tumor therapies. Their shape and size are two of the key factors for a successful treatment. Indeed, the unique properties of the star-shaped NPs have made it possible to enhance the latter one with respect to other forms such as nanorods, nanospheres, nanoprisms, etc. The large extinction cross section and the anisotropy of the AuNPs make the interaction with the electromagnetic waves stronger, thereby allowing the use of NIR, in which biological tissues are usually almost transparent. In regard to the dimensions, it has been observed that the small AuNPs can be more efficiently retained in the tumor tissues thanks to the EPR effect, guaranteeing greater diagnostic and/or therapeutic efficiency. Moreover, they can both be expelled more easily and be capable of exceeding the BBB in the case of brain tumors. A better understanding of the behavior and properties of ultra-small AuNPs in different tumors can broaden their biomedical application.

To date, experimentation on functionalized AuNPs as a contrast tool in various imaging techniques is becoming more important. Contrary to traditional contrast agents, their high atomic number is essential in computed tomography, allowing for clearer visualization of the tumor site. Furthermore, the important aspects are their non-toxicity and excretion through the kidneys.

As stated above, AuNPs absorb easily in the NIR range. This allows researchers to improve the diagnostic imaging techniques such as photoacoustic imaging, in which the same photoacoustic effect is exploited.

As far as diagnostics is concerned, the NIR absorption characteristic helps photothermal therapy by converting this electromagnetic wave into heat. This provides the heating of the involved tissues up to temperatures between 41 °C and 47 °C, thus facilitating cell death.

In photodynamic therapy, AuNPs assume the role of photosensitizers, which, following irradiation, produce ROS, whose goal is to kill cancer cells.

Last but not least, the improvement of radiotherapy has also been described. Here too, suitably functionalized AuNPs promote treatment efficiency thanks to both their high atomic number and their ability to reduce the radiation dose to healthy tissues, thus preserving them.

Although numerous results demonstrating the efficiency of AuNPs in the medical field have been described and reported, there is still a long way to go before we can use them in humans. One of the main questions is the damage that these NPs can cause in the long term, years after their first use in the human body. Furthermore, the innumerable tumor differences could require different AuNPs. In addition, the aspect concerning the variability of the tumors in each analyzed subject is not negligible. 

Today, ongoing experimentation is focused on understanding the effect of AuNPs on countless types of tumors to create a smart Au nanoplatform for cancer treatment by tuning the size, shape and concentration and finding tumor-specific functionalizing agents with therapeutic properties. The goal is to develop a personalized nanomedicine treatment that is specific to each patient by analyzing the single case, ensuring the low toxicity behavior of the NPs. 

In the diagnostics field, we think that the introduction of AuNPs as a contrast agent with low toxic effects will be easier to achieve in the future. We are therefore confident that medicine for cancer treatments will undergo a great improvement in the coming years using Au nanomaterials.

## Figures and Tables

**Table 1:** Melting points of AuNPs for different nanoparticle size measured by Dick et al. [25] and Liu et al. [17,26].

Size Range	Melting Point
5 nm	830 °C
2 nm	350 °C
1 nm	200 °C

## Data Availability

Not applicable.
